# Biomethane potential of industrial paper wastes and investigation of the methanogenic communities involved

**DOI:** 10.1186/s13068-016-0435-z

**Published:** 2016-01-26

**Authors:** Andreas Walter, Sandra Silberberger, Marina Fernández-Delgado Juárez, Heribert Insam, Ingrid H. Franke-Whittle

**Affiliations:** Institut für Mikrobiologie, Universität Innsbruck, Technikerstraße 25d, 6020 Innsbruck, Austria; Hochschule Hamm-Lippstadt, Biotechnologie, Marker Allee 76-78, 59063 Hamm, Germany

**Keywords:** Pulp and paper residues, Anaerobic digestion, Pretreatments, ANAEROCHIP microarray, qPCR, Methanogens, *Methanosarcina*

## Abstract

**Background:**

Cellulose-containing waste products from the agricultural or industrial sector are potentially one of the largest sources of renewable energy on earth. In this study, the biomethane potential (BMP) of two types of industrial paper wastes, wood and pulp residues (WR and PR, respectively), were evaluated under both mesophilic and thermophilic conditions, and various pretreatment methods were applied in the attempt to increase the methane potential during anaerobic digestion. The methanogenic community composition was investigated with denaturing gradient gel electrophoresis (DGGE) and the ANAEROCHIP microarray, and dominant methanogens were quantitated using quantitative PCR.

**Results:**

All pretreatments investigated in this study with the exception of the alkaline pretreatment of PR were found to increase the BMP of two paper industry wastes. However, the low recalcitrance level of the PR resulted in the pretreatments being less effective in increasing BMP when compared with those for WR. These results were supported by the physico-chemical data. A combined application of ultrasound and enzymatic pretreatment was found to be the best strategy for increasing methane yields. The retention time of substrates in the reactors strongly influenced the BMP of wastes subjected to the different pretreatments. In sludges from both paper wastes subjected to the various pretreatments, mixotrophic *Methanosarcina* species were found to dominate the community, accompanied by a consortium of hydrogenotrophic genera.

**Conclusions:**

Pretreating industrial paper wastes could be a potentially viable option for increasing the overall degradation efficiency and decreasing reactor retention time for the digestion of complex organic matter such as lignocellulose or hemicellulose. This would help reduce the environmental burden generated from paper production. Although there were minor differences in the methanogenic communities depending on the temperature of anaerobic digestion, there was little effect of substrate and pretreatment type on the community composition. Thus, methanogen community dynamics would not seem to be an appropriate indicator regarding BMP in the AD processes investigated.

## Background

Due to the incessant and escalating demand and consumption of paper-based products, the pulp and paper market is one of the world’s fastest growing industries, expected to increase by 60 % between 2012 and 2020 [1]. Paper, as we know it today, was developed in China almost 2000 years ago [2], and there are five basic steps in the process of pulp and paper production. In a preliminary debarking step, bark is removed and the wood is converted into smaller wooden chips. Pulping (mechanical or chemical) follows debarking, and in this step the majority of lignin and hemicellulose is removed from wooden chips. The brown pulp is then bleached such that the desired colour (dictated by product standards) is obtained. Bleaching agents are removed in a subsequent washing step [3], and the pulp slurry generated is subsequently dried on a paper machine and sheets of paper are produced.

The pulp and paper industry is, however, a major consumer of natural resources (wood and water) as well as energy (fossil fuels and electricity), and typically produces significant amounts of environmentally damaging pollutants [2]. Because of increasing legislative pressures forcing the industry to ‘clean-up’, as well as advances in process technologies, the industry has managed to reduce its impact on the environment during recent decades by 80–90 % [2].

The anaerobic digestion (AD) of residues arising from the production of paper has the potential to concurrently counteract environmental and economic issues. In a cascade of steps including hydrolysis, acidogenesis, acetogenesis and methanogenesis, a consortium of bacteria and archaea convert organic matter into biogas (50–80 % methane), an energy carrier [4]. However, in the case of pulp and paper wastes, the inherent recalcitrant nature of the lignocellulosic materials can result in a problematic bioconversion to biogas. The AD of such materials is limited by the rate of hydrolysis, because the primary biodegradable polymer, cellulose, is shielded by both lignin and hemicellulose [5]. This complex structure dictates that the degradation process occurs slowly, and thus long hydrolytic retention times and reactor volumes are required for the large-scale AD of such wastes, resulting in higher capital costs [6].

In order to increase the overall degradation efficiency and decrease reactor retention time for the digestion of complex organic matter such as lignocellulose or hemicellulose, different pretreatment methods can be applied [4, 7]. Different physical, chemical, physico-chemical and biological pretreatments have been used in various studies to break plant cell wall structures, making the organic materials more susceptible to hydrolysis, and thus aiding the AD process [8]. However, no studies are known to the authors where different pretreatment strategies have been applied to solid paper wastes and their impact on the biomethane potential (BMP) have been analysed. Nothing is known about the influence of the various pretreatments on methanogenic communities in such wastes.

The aim of this study was to determine the BMP of two industrial paper wastes. In order to assess the BMP, two different temperatures for AD were tested (37 and 55 °C), as were six different pretreatment methods. Pretreatment methods included an autoclave pretreatment, an ultrasound pretreatment, an alkali (NaOH) pretreatment, the use of enzymes (cellulases, hemicellulases, ligninases), a combination of enzymes with ultrasound pretreatment, and the addition of commercially available hydrolytic microorganisms.

It is hypothesised that waste-derived methane yields could be increased by various pretreatments. A second hypothesis was that the pretreatments would exert little influence on the methanogenic communities in the resulting anaerobic sludges.

In order to investigate the effects of the different pretreatments and AD temperatures on the methanogenic communities involved, denaturing gradient gel electrophoresis (DGGE) and ANAEROCHIP microarrays combined with quantitative PCR (qPCR) were applied.

## Results and discussion

### Physico-chemical parameter analysis

Physico-chemical properties of the inoculum cattle slurry (CS) and industrial paper wastes pulp residues (PR) and wood residues (WR) are shown in Table 1. The same properties for the sludges obtained after 23 days of AD of the waste materials subjected to various pretreatments are shown in Table 2.

**Table 1:** Physico-chemical characteristics of inoculum and paper wastes

	Cattle slurry	Wood residues	Pulp residues
TS (% FW w/v)	8.23 (0.18)	36.5 (0.06)	41.0 (0.08)
VS (% FW w/v)	5.82 (0.13)	35.3 (0.07)	23.3 (0.06)
pH	7.95 (0.02)	2.31 (0.02)	8.55 (0.02)
NH_4_-N (mg L^−1^)	1600 (159)	nm	nm
Cellulose content (%)	nm	44.6	46.3
Hemicellulose content (%)	nm	1.20	4.50
Lignin content (%)	nm	11.4	0.03

*n* = 3; standard error is given in brackets

*TS* total solids, *FW* fresh weight, *VS* volatile solids, *nm* not measured

**Table 2:** Physico-chemical parameters of sludges measured after 23 days of anaerobic digestion

Name	TS (% FW w/v)	VS (% FW w/v)	pH	TAN (mg L^−1^)	Acetate (mM)	Propionate (mM)	CH_4_ (Nml gVS^−1^)
WR37	7.74 (0.13) a	5.44 (0.08) a	7.81 (0.02) a	1533 (44.1) ab	0.13 (0.13)	nd	116 (7.51) a
WR37au	7.34 (0.07) ac	5.27 (0.05) a	8.16 (0.02) b	1867 (136) b	nd	nd	165 (2.08) ab
WR37us	6.35 (0.07) b	4.53 (0.05) b	8.04 (0.01) b	1250 (50.0) a	0.04 (0.04)	nd	195 (4.42) ab
WR37al	6.10 (0.08) bd	4.17 (0.07) d	8.11 (0.01) b	1350 (132) a	0.57 (0.46)	0.25 (0.25)	139 (12.4) ab
WR37mo	6.95 (0.14) c	4.86 (0.06) c	7.82 (0.04) a	1367 (183) ab	nd	nd	137 (36.2) ac
WR37en	6.09 (0.18) bd	4.20 (0.08) d	7.79 (0.06) a	1433 (44.1) ab	0.10 (0.05)	nd	203 (23.3) bc
WR37us+en	5.63 (0.04) d	3.99 (0.03) d	7.64 (0.01) c	1417 (60.1) ab	nd	nd	222 (5.04) b
PR37	7.87 (0.02) a	4.74 (0.03) a	7.82 (0.02) a	1800 (50.0) a	0.12 (0.07)	nd	323 (4.96) a
PR37au	7.61 (0.26) a	4.67 (0.21) a	8.15 (0.02) b	1683 (174) ab	nd	nd	331 (8.53) a
PR37us	7.25 (0.25) a	4.62 (0.13) a	8.00 (0.01) c	1650 (104) ab	0.03 (0.03)	nd	400 (7.30) b
PR37al	6.06 (0.16) bc	3.57 (0.13) b	8.06 (0.02) c	1567 (267) ab	0.49 (0.24)	1.17 (0.62)	202 (17.8) c
PR37mo	7.17 (0.07) a	4.23 (0.05) ac	7.79 (0.01) a	1567 (44.1) ab	0.07 (0.04)	nd	324 (5.73) a
PR37en	6.38 (0.05) b	3.81 (0.02) bc	7.66 (0.01) d	1500 (50.0) ab	0.03 (0.03)	nd	351 (2.79) a
PR37us+en	5.54 (0.03) c	3.41 (0.03) b	7.59 (0.02) e	1067 (16.7) b	nd	nd	408 (8.37) b
WR55	3.81 (0.30)	2.48 (0.22)	8.30 (0.03)	1550 (76.4)	nd	1.65 (1.30)	171 (6.01)
PR55	2.91 (0.02)	1.77 (0.05)	8.31 (0.04)	1567 (44.1)	1.11 (0.35)	6.75 (0.66)	368 (7.89)

*n* = 3; standard error is given in brackets. Different letters (a–e) indicate significant differences between pretreatments (*p* < 0.05) for each waste material according to the Tukey HSD test

*TS* total solids, *VS* volatile solids, *FW* fresh weigh, *nd* not detected, *WR* wood residues, *PR* pulp residues, *37* AD at 37 °C, *55* AD at 55 °C, *au* autoclave pretreatment, *us* ultrasound pretreatment, *al* alkaline pretreatment, *mo* pretreatment with commercial microorganism mixture, *en* enzyme pretreatment

As expected, there was a reduction in the total solids (TS) and volatile solids (VS) in all samples after AD. The lowest TS and VS values were found in sludges in which the wastes were pretreated with a combination of enzymes and ultrasound. Lower TS and VS levels were also revealed after AD at 55 °C (WR55 and PR55) compared with after AD at 37 °C. Together, these results indicate a higher organic carbon mineralisation into methane under thermophilic conditions.

The highest pH values were found in the sludges from AD at 55 °C (WR55 and PR55), while significantly lower values were observed in the sludges where wastes were pretreated with ultrasound and enzymes (WR37us+en, PS37us+en). Higher pH values under thermophilic conditions have been observed previously and explained by the bioenergetics balance and alkalinity differences [9, 10].

Total ammonia nitrogen (TAN) concentrations increased during AD as a result of urea and protein hydrolysis, but were not found to be inhibitory. No clear differences were observed between mesophilically and thermophilically treated samples. Results are in line with the findings of Moset et al. [10].

Acetate levels were low (<0.57 mM) in all mesophilic sludges. Under thermophilic conditions however, a higher acetate concentration (1.11 mM) was found in the PR, although no acetate was detected in WR. Propionate was detected only in the mesophilic sludges treated under alkaline conditions (al), while it was detected at higher levels in both thermophilic sludges investigated. Similar observations were reported by Moset et al. [10] and Kim et al. [11]. Nonetheless, all acetate and propionate concentrations measured were found to be far below the critical values of 45 and 15 mM, respectively [12]. Levels of butyrate, isobutyrate, valerate and isovalerate, suggested to be good indicators for reactor instability [13], were also found to be under the detection limit of 0.5 mM (results not shown). The low concentrations of volatile fatty acids (VFA) in the samples after AD indicate an efficient transformation of metabolites into CH_4_ through a well-balanced consortium of bacteria and methanogens during the AD process.

### Evaluation of pretreatment efficiency to increase methane yield

The degradation of complex polymers is known to be a bottleneck in AD processes [7]. Hemicellulases and cellulases produced by the bacterial digester community are capable of the breakdown of hemicellulolytic polymers into mono-, di-, and oligosaccharides. Nevertheless, the degradation efficiency is low. Hemicellulose consists mostly of heteroxylans (hardwood hemicellulose) or glucomannans (softwood hemicellulose), connected through diferulic or isodityrosine bridges and forming an insoluble network, in which cellulose microfibrils are imbedded [14]. Thus, increasing the hydrolysis rate of these complex polymers by some form of pretreatment is crucial in engineered AD processes in order to improve the biomass-conversion efficiency and decrease the hydraulic retention time [4].

**Fig. 1:**
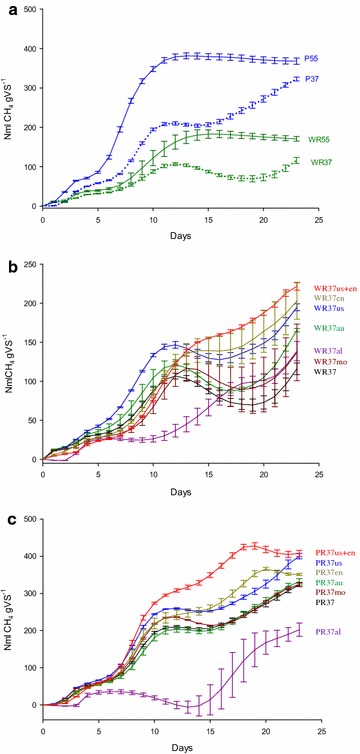
**a** Accumulated methane production from non-pretreated waste residues (WR) and pulp residues (PR) at 37 °C and at 55 °C. Average values (*n* = 3) are plotted and *error bars* indicate standard error. **b** Accumulated methane production at 37 °C from pretreated wood residues (WR). *au* autoclave pretreatment, *us* ultrasound pretreatment, *al* alkaline pretreatment, *mo* commercial microorganism mixture pretreatment, *en* enzyme pretreatment. Average values (*n* = 3) are *plotted* and *error bars* indicate standard error. **c** Accumulated methane production at 37 °C from pretreated pulp residues (PR). Average values (*n* = 3) are *plotted* and *error bars* indicate standard error

In this study, pretreatment efficiency was evaluated by comparing the daily and accumulated CH_4_ yield of both pretreated and non-pretreated paper wastes during a 23-day AD process. Non-pretreated WR and PR produced 116 (± 7.51) and 323 (± 4.96) ml CH_4_ g^−1^ VS at 37 °C, and 171 (± 6.01) and 368 (± 7.89) ml CH_4_ g^−1^ VS at 55 °C, respectively (Table 2 and Fig. 1a). The lower CH_4_ yields from WR compared with PR may be attributed to the higher lignin content (11.4 %, Table 1). Results are in line with yields from paper wastes published by Raposo et al. [15], where yields of 120–320 ml CH_4_ g^−1^ VS for pulp and 84–369 ml CH_4_ g^−1^ VS for paper waste were reported. Temperature did not affect methane production (p = 0.17), but yields obtained from PR were significantly higher than result obtained from WR according to the Mann–Whitney tests (p < 0.01).

All pretreatments of WR resulted in CH_4_ yield increases (Table 2; Fig. 1b). In contrast, there was a significant decrease in CH_4_ yield (37 %) after alkaline pretreatment of PR, and only after pretreatment with ultrasound and a combination of ultrasound and enzymes were significant increases in CH_4_ production from PR obtained. The lower BMP resulting from alkaline treatment is most likely due to a negative effect on the microbial community imposed by sodium ions, dissociated from NaOH. Na ions at low concentrations are essential for microorganisms, probably because of their role in the formation of adenosine triphosphate or the oxidation of NADH [16]. Moderate Na concentrations have been found to stimulate microbial growth and to be antagonistic to ammonia inhibition [17, 18]. Excessive Na amounts, however, can decrease and inhibit growth [19]. Soto et al. [20] and Liu and Boone [21] detected toxic effects of Na on VFA and lignocellulose degrading bacteria as well as on methanogens at concentrations of 14 and 27.7 g L^−1^, respectively. According to the literature, the level at which Na exerts a toxic effect varies, probably due to methodological and environmental differences and microbial adaptation to particular conditions. It would seem that the 10 g L^−1^ NaOH used in this study caused a negative effect on the AD of PR, which most likely absorbed the NaOH. Such an effect was not observed for the WR. A detoxification of the PR waste prior to AD by removal of the NaOH possibly would have resulted in improved methane yields. According to the literature, other alkaline agents such as KOH or lime could be used for pretreatment. However, according to Zhu et al. [22] and Penaud et al. [23], the use of NaOH revealed the highest pretreatment efficiency of all tested bases.

Acetate levels were higher in sludges of both WR and PR subjected to alkaline pretreatment, when compared to all other treatments (Table 2) and propionate was exclusively detected in both wastes which underwent alkaline pretreatment, with average concentrations up to 1.17 mM (PR37al). These findings indicate a negative effect on both propionate and acetate utilising microorganisms, and are in line with the results of Soto et al. [20].

Of the two physical disintegration methods applied in this study, ultrasound was found to be the most efficient in raising the BMP. A 68 % increase in CH_4_ yield for WR and a 24 % increase in methane yield for PR were found after 23 days of AD, when compared to non-pretreated wastes. Positive effects of ultrasound pretreatment have been published before. In a study of Khanal et al. [24], a digester receiving sonicated waste activated sludge removed 11–39 % more soluble COD than the digester receiving non-sonicated sludge. Also, Muller et al. [25] reported an improvement of gas production by 17 %, with a 6.2 % increase in total solids removal in mesophilic 38 L reactors with 15 days of retention times.

However, disadvantages with this pretreatment approach exist, due to the high amount of energy required and technical issues in large-scale applications. The autoclaving pretreatments were found to be less effective, resulting in a 43 % CH_4_ increase for the WR, and a negligible 3 % increase for PR.

The effectiveness of amendment with microorganisms was lower for both WR and PR compared with other methods (except for alkaline treatment of PR). There was an 18 % increase in CH_4_ production in WR37mo, while there was no increase in CH_4_ yield for PR37mo. It is possible that the 23 days of AD used in the experiment was not long enough to allow an efficient adaption of the amended microorganisms to the reactor conditions. The application of commercially available enzymes as a pretreatment approach resulted in a significant increase in CH_4_ production for WR (+75 %), but in only a small increase for PR (+9 % CH_4_). Most likely, the enzymes were able to aid in the degradation of the lignocellulosic WR, but were not as useful with the pulp waste which had lower lignin content. Mayhew et al. [26] were able to increase the biogas production by 10 %, when pretreating waste activated sludge with enzymes at 42 °C for 2 days. In the study of Davidsson et al. [27], methane production was increased by 60 % during pilot-scale trials, by introducing prepared enzyme solution to a pre-hydrolysation contact chamber with an HRT of 4 h.

As stated by Parawira [28], pretreating pulp and paper residues with microorganisms or cell-free enzymes can be effective if the appropriate microorganisms or enzymes are applied and operating conditions, dosage and enzyme-to-waste ratio are optimal. The applications of both microorganisms and cell-free enzymes have advantages and disadvantages. Cell-free enzymes, on the one hand, are active under a wide range of environmental conditions and remain active even when conditions quickly change [28]. Enzymes also tolerate the presence of different microorganisms and possible inhibitors of microbial metabolism. In contrast, the implementation of vital microorganisms is potentially more dynamic and efficient, due to the ability of microorganisms to directly produce enzymes as well as their physiological flexibility and motility. However, in this study, the bacterial consortium selected was found to be incapable of increasing the degradation efficiency of PR. Results indicated that the application or pretreatment conditions used were either inadequate or inappropriate.

The combined application of ultrasound and enzymes as a pretreatment method was found to be the most suitable approach for both wastes. Significantly higher CH_4_ yields of +91 and +26 % were revealed for WR37us+en and PR37us+en, respectively, compared to non-pretreated wastes after 23 days of AD. Pretreatment with ultrasound waves most likely increased the accessible surface area and decreased the polymerisation degree of the cellulose. As a result, enzymes could attack the disintegrated fibres more efficiently, due to the enlarged surface area.

The residence time of substrates in the AD reactors was found to strongly influence the BMP of paper wastes subjected to the different pretreatments (Figs. 1b, 1c). After 7 days of AD, WR subjected to an autoclaving pretreatment (+23 % CH_4_) or ultrasound pretreatment (+61 % CH_4_) yielded the highest amounts of CH_4_. Compared to non-pretreated WR, enzymes alone (−12 % CH_4_) or in combination with ultrasound pretreatment (−27 % CH_4_) revealed lower CH_4_ yields. In contrast, after 14 days of AD, the increase in CH_4_ yield for autoclaving (+12 %), ultrasound (+40 %) and enzyme pretreatments (+44 %) were lower than that of the combined ultrasound and enzyme pretreatment (+55 %). Pretreatment with ultrasound alone, and in combination with enzymes, were found to be the most suitable methods for enhancing CH_4_ yields of PR under both short and long reactor residence times. Increases of 30 and 27 % for the ultrasound and ultrasound/enzyme treatments, respectively, were reported after 7 days of AD. After 14 days, CH_4_ yields increased in the combined pretreatments compared to non-pretreated PR (+61 % CH_4_), but decreased in ultrasound pretreatments (+23 % CH_4_). These findings illustrate the importance of striking a balance between shortened AD times and efficient substrate degradation in biogas plant operation.

Methane production curves (Fig. 1a–c) revealed the existence of multiple phases under mesophilic conditions. Such diauxic phenomena are frequently observed in batch experiments when high concentrations of homologous substrates are processed [29]. Under such conditions, the substrate which supports the highest growth rate is utilised initially, while the consumption of poorer substrates remains repressed. Diauxie has also been observed in reactors digesting multi-component agro-wastes [30] and cow manure with food waste or leaves/straw [31]. Kübler and Schertler [32] observed an initial inhibition of the hydrolysis of cellulosic material when dissolved and easy-to-degrade monomers such as sugars were present. The polymeric structural carbohydrates were degraded subsequently in a second hydrolysation phase. We suppose the same phenomena appeared in this study and resulted in the biphasic or multiphasic CH_4_ production curves. Also, short-time decreases in methane yields can be observed in several of the graphs showing cumulative methane yield under mesophilic conditions. This effect may be attributed to metabolic adjustments in-between phases and an improved degradation of the CS at that point in time. Thus, when subtracting the negative control CS from the treatments, it appeared that a decrease in the cumulated methane amount occurred.

### Methanogen community composition and stability

**Fig. 2:**
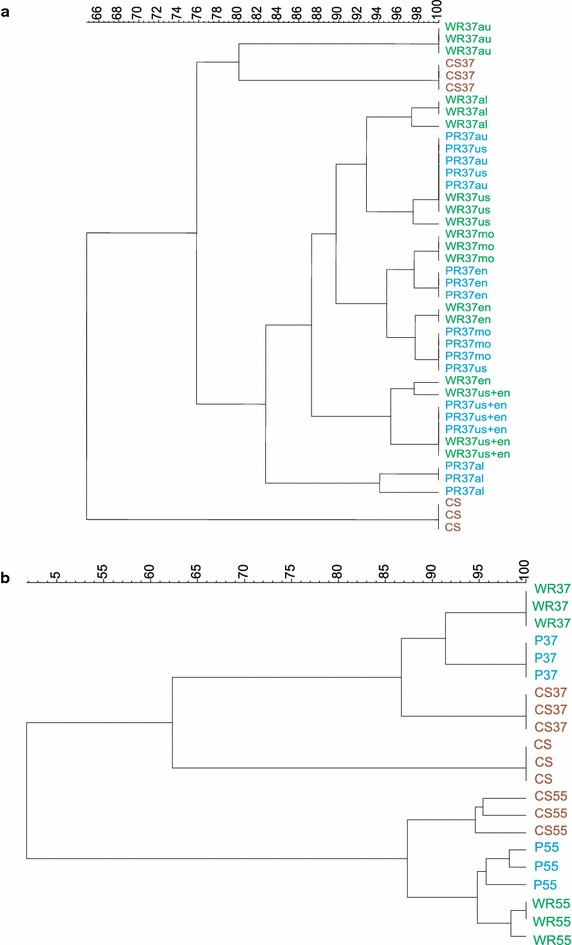
**a** Cluster analysis of DGGE fingerprints based on the PCR of archaeal *16S rRNA* genes extracted from undigested cattle slurry (CS) and sludges after anaerobic digestion at 37 °C. *WR* Wood residues, *PR* Pulp residues, *37* Anaerobic digestion at 37 °C, *au* autoclave pretreatment, *us* ultrasound pretreatment, *al* alkaline pretreatment, *mo* commercial microorganism mixture addition, *en* enzyme pretreatment. **b** Cluster analysis of DGGE fingerprints based on the PCR of archaeal *16S rRNA* genes extracted from cattle slurry and sludges after anaerobic digestion at 37 and 55 °C. *CS* cattle slurry, *WR* wood residues, *PR* pulp residues, *37* anaerobic digestion at 37 °C, *55* anaerobic digestion at 55 °C

DNA from CS and sludges after AD at 37 °C were subjected to DGGE and bands were analysed in a fingerprint cluster analysis (Fig. 2a). The undigested CS was found to group distinctly from all samples, while PR and WR subjected to different pretreatments showed more than 75 % similarity to each other. Digestates did not, however, group together according to waste origin or pretreatment type; rather, a large cluster containing all samples except the non-pretreated CS and the WR subjected to autoclaving was observed. Samples in this cluster shared more than 80 % similarity with each other.

By comparing anaerobic sludges treated at mesophilic and thermophilic temperatures, a clear effect on the archaeal communities was detected after 23 days of AD (Fig. 2b). The CS not subjected to AD grouped distinctly from both the 37 and 55 °C digestates, but more closely with the 37 °C digestates (62.5 % similarity). The WR and PR paper wastes were found to have no significant influence on the fingerprinting patterns obtained after AD.

**Fig. 3:**
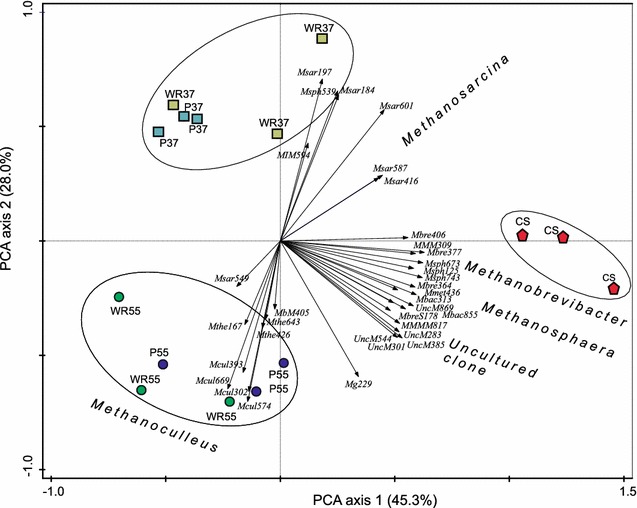
Loading plot obtained by principal component analysis, depicting the clustering of cattle slurry and the digestates WR37, PR37, WR55 and PR55. The vectors represent the different probes of the ANAEROCHIP microarray. *CS* cattle slurry, *WR* wood residues, *PR* pulp residues, *37* anaerobic digestion at 37 °C, *55* anaerobic digestion at 55 °C

To investigate the methanogen communities in the digestates of WR37, PR37, WR55, PR55 and CS, the *16S rRNA* gene-based ANAEROCHIP microarray was applied. In all samples, signal-to-noise ratios (SNRs) >2 were found for probes targeting the family *Methanobacteriaceae* (family to which genus *Methanobrevibacter* belongs) as well as the genera *Methanosarcina*, *Methanobrevibacter*, *Methanoculleus* and *Methanosphaera* (results not shown). Additionally, probes targeting an uncultured clone [33] hybridised with DNA from the CS and sludges after AD at thermophilic conditions (WR55 and PR55). SNRs exceeding the threshold level of two were included in a PCA (Fig. 3). The two axes represent 73.3 % of the variance, with the first and second axis representing 45.3 and 28.0 % of the variance, respectively. Different samples are represented by circles, squares and polygons, the oligonucleotide probes of the ANAEROCHIP microarrays by vectors. Particular probes can be seen to be more important in discriminating the samples. The lengths of the arrows indicate the significance for sample differentiation, and arrows point in the direction of samples with above average signal. Similar vector direction of probes signifies high covariance, indicating probes were detected mutually in the samples.

The PCA loading plot clustering supports the findings of DGGE fingerprinting, whereby the undigested CS clustered distinctly to the samples after 23 days of AD. Additionally, wastes treated under mesophilic conditions were distinct from wastes digested under thermophilic conditions.

qPCR was conducted to quantify methanogens (with the exception of the uncultured clone for which no positive control was available) detected with the ANAEROCHIP microarray (Table 3). In all samples investigated, *Methanosarcina* was the most dominant genus. Despite the non-significant difference, it is worth mentioning the increase in the gene copy number (GCN) of *Methanosarcina* found in WR and PR samples digested under mesophilic temperatures (2.39 × 10^8^ and 2.03 × 10^8^, respectively) when compared to the inoculum CS (5.80 × 10^7^). In contrast, there was a slight, but significant decrease in the GCN of *Methanosarcina* in the WR55 and PR55 samples (2.48 × 10^7^ and 4.75 × 10^7^, respectively) compared to inoculum CS.

**Table 3:** Primers, standard curve parameters and number of gene copies ml^−1^ sample of the investigated genera

	*Methanosarcina*	*Methanobrevibacter*	*Methanoculleus*	*Methanosphaera*
F primer	240F (CCTATCAGGTAGTAGTGGGTGTAAT)	210F (TTTCGCCTAAGGATGGGTCT)	298F (GGAGCAAGAGCCCGGAGT)	594F (TAAGTCTTTGGTGAAAGCTT)
R primer	589R (CCCGGAGGACTGACCAAA)	367R (CGATTTCTCACATTGCGGAG)	586R (CCAAGAGACTTAACAACCCA)	747R (GTTACTCACCGTCAAGAT)
*R* ^*2*^ value	0.9999	0.9993	0.9999	0.9994
Slope	−4.53	−3.958	−4.679	-4.358
Intersept	38.913	35.106	43.209	38.283
Efficiency	0.66	0.79	0.64	0.70
CS	5.80E+07 (3.24E+06) a	2.99E+07 (3.98E+06) a	1.98E+06 (8.95E+05) a	2.31E+06 (2.90E+05) a
WR37	2.39E+08 (2.98E+07) ab	2.13E+07 (3.29E+06) ab	4.63E+06 (9.85E+05) ab	2.95E+06 (7.85E+05) ab
WR55	2.48E+07 (4.53E+06) b	1.21E+07 (1.90E+06) b	9.51E+06 (1.74E+06) b	7.66E+05 (1.98E+05) b
PR37	2.03E+08 (1.71E+07) a	2.62E+07 (2.55E+06) a	1.04E+07 (1.63E+06) a	2.91E+06 (6.09E+05) a
PR55	4.75E+07 (3.28E+06) b	1.21E+07 (1.28E+06) b	1.95E+07 (6.18E+06) b	7.83E+05 (1.51E+04) b
WR37us+en	1.67E+08 (2.06E+07) b	2.09E+07 (3.56E+06) ab	7.23E+06 (5.46E+05) ab	2.11E+06 (1.23E+05) ab
PR37us+en	1.54E+08 (1.85E+07) ab	3.40E+07 (4.48E+06) a	1.12E+07 (2.98E+06) a	7.15E+05 (9.79E+04) a

*n* = 6; standard error is given in brackets. Different letters indicate significant differences between pretreatments (*p* < 0.05) according to the Tukey HSD test

*CS* cattle slurry, *WR* wood residues, *PR* pulp residues, *37* anaerobic digestion at 37 °C, *55* anaerobic digestion at 55 °C, *us* ultrasound pretreatment, *en* enzyme pretreatment

Dominance of *Methanosarcina* has been frequently reported in the literature for reactors treating manures [10, 34, 35] and reactors experiencing instable conditions during start-up procedures [33, 36]. *Methanosarcina* is capable of metabolising a broad spectrum of substrates, including H_2_, CO_2_, methanol, methylamine and acetate [37]. Being capable of both acetoclastic and hydrogenotrophic methanogenesis (i.e. mixotrophic methanogenesis), *Methanosarcina* is more flexible to changing substrate conditions and the presence of inhibitors [38]. It is characterised by high growth rates (i.e. doubling times in the order of 1–1.2 days) and a tolerance to sudden changes in pH of around 0.8–1.0 units. In comparison, other methanogens have doubling times of 4–6 days and tend to be affected by pH changes of 0.5 units or even less [39, 40]. The ability of *Methanosarcina* to acclimatise and adapt more effective to modified environmental conditions and to dominate the methanogenic community in our study was therefore not surprising.

*Methanobrevibacter* was found to be the second most prevalent methanogenic genus in all samples, with GCNs of >10^7^. This strictly hydrogenotrophic genus is considered to be the dominant methanogen in the rumen environment, representing up to 61.6 % of the archaeal community [41] due to its high growth rate and ability to competitively utilise H_2_ and CO_2_ [42]. Consequently, the detection of high *Methanobrevibacter* numbers in the CS samples of this study was not unexpected and has been confirmed in other studies [33, 43, 44]. GCNs of the genus in samples digested under thermophilic conditions (PR55 and WR55) were significantly lower than in the CS control (Table 3).

In contrast, a significant increase of *Methanoculleus* in GCN was detected after AD under thermophilic conditions for both waste residues. GCNs of the hydrogenotrophic methanogen *Methanosphaera* were found to remain stable during mesophilic AD of WR and PR wastes (~2 × 10^6^), but decreased approximately fivefold in samples after AD under thermophilic conditions (Table 3). This is most likely because no thermophilic species are known to exist in the genus *Methanosphaera* [37].

Due to the relatively low level of variation in numbers of the dominant methanogens investigated using qPCR, it would appear that the quantity of these methanogens in samples cannot be used as an appropriate indicator for BMP in the AD processes investigated. The role of acetate oxidisers in this study, with respect to the generation of CH_4_, is unclear. Westerholm et al. [45] found *Methanosarcina* and syntrophic acetate-oxidising bacteria in high abundance in mesophilic reactors, fed with a mixture of cattle manure and silage. This indicated that the genus acted as an important hydrogenotrophic, and not acetoclastic methanogen in the reactors investigated. Thus, it would appear that this mixotrophic methanogen can act as a mediator of the whole acetate-oxidation process, as proposed previously [46].

## Conclusions

This study has shown that all pretreatments investigated were found to considerably increase the methane yields from the WR. Pretreatment of the PR was found to be less effective in increasing BMP, due to its low recalcitrance. These results were supported by the physico-chemical data. The combined application of ultrasound with enzymes was the best possible pretreatment strategy for both PR and WR wastes. Chemical and biological pretreatments, more cost-effective approaches than ultrasound and enzymatic pretreatments [8], revealed differing results for the two wastes. Pretreatment with microorganisms and enzymes was found to be a suitable approach for WR, but less effective for PR. The application of NaOH was not effective at all, probably due to inhibition from the high sodium ion concentrations.

The retention time of substrates in the reactors strongly influenced the BMP of wastes subjected to the different pretreatments. This finding illustrates the importance of finding a balance between shortened retention times and efficient substrate degradation in biogas plant operation.

In all substrate-amended reactors, *Methanosarcina* was the dominating genus, accompanied by a consortium of hydrogenotrophs. However, relatively little variation in the numbers of the dominant methanogens were found in digested sludges of the wastes subjected to various pretreatments prior to AD, using qPCR. Thus, methanogen numbers would not seem to be an appropriate indicator regarding BMP in the AD processes investigated.

In conclusion, it would seem that the AD of pretreated pulp and paper wastes could be a potentially viable option for utilising and reducing the huge amounts of waste products generated from one of the world’s biggest industries. This would help reduce the environmental burden generated from paper production. More research, however, is needed in order to further investigate the economical issues involved with pretreatment of the wastes.

## Methods

### Paper wastes and inoculum source for AD

The two pulp and paper wastes tested in this study were WR, produced in the mechanical and chemical pulping process and bleached PR, collected after screening, cleaning and washing of the pulp product. To decrease particle size to below 2 mm, WR and PR were ground in a coffee grinder (CG100 140 W; De’Longhi–Kenwood, Hampshire, United Kingdom). Cellulose, hemicellulose and lignin contents in WR and PR were determined and calculated as described by Van Soest et al. [47]. CS collected from a farm in Innsbruck (Tyrol, Austria; 47° 16′ 2″ N, 11° 23′ 34″ E) was used as an inoculum for AD. CS was sieved with a 4-mm sieve and left over night at 37 °C to degas. Test materials and inoculum were stored in aliquots at −20 °C until use. Substrate and inoculum characteristics are listed in Table 1.

### Pretreatment of paper wastes

WR and PR were subjected to six different pretreatments. For thermal-high pressure extraction, residues were autoclaved (au) at 121 °C with 1 bar overpressure in a steam steriliser (Varioklav, HP Medizintechnik GmbH, Oberschleißheim, Germany) for 20 min. An ultrasound (us) pretreatment was conducted by adding 50 ml distilled water to 15.8 g WR and 23.5 g PR, and subjecting residues to ultrasound wave frequencies (Elmasonic S 100 H, Elma, Schmidbauer GmbH, Singen, Germany) at 37 kHz and 600 W for 30 min. For the alkaline pretreatment (al), 15.84 g WR and 23.5 g PR were submerged in 100 ml of 1 % NaOH (w/v). Flasks were incubated at room temperature for 24 h, after which time the NaOH-containing supernatant was carefully removed. An enzyme (en) pretreatment was conducted by adding 4.8 µl of an enzyme mix containing equal amounts of cellulases, hemicellulases and ligninases (Novozymes, Franklinton, United States) to 15.84 g WR and 23.5 g PR suspended in 50 and 75 ml of distilled water, respectively. After pH adjustment to six, flasks were incubated at 55 °C with shaking (125 rpm) for 24 h. Thereafter, enzymes were inactivated by boiling pretreated residues for 15 min. Additionally, a combination of an ultrasound and enzyme pretreatment (us+en) was conducted. Finally, 6 × 10^−4^ g BGMax 3000 commercial microorganism mix (mo) obtained from Novozymes (Franklinton, United States) was added to reactor flasks directly prior to AD, and additionally, on days 7 and 14. BGMax 3000 contains viable bacterial, fungal and yeast cultures [48].

### Determination of BMP

To determine methane production from wastes at different temperatures and to compare the efficiency of the various pretreatments, a BMP test was performed in a batch-mode AMPTS II (Bioprocess Control, Lund, Sweden) for 23 days. Treatments were tested in triplicate in total reactor volumes of 400 ml. The inoculum-to-substrate ratios (CS:WR and CS:PR) calculated with VS amounts were set to four. Non-pretreated WR and PR were digested under mesophilic (37 °C) and thermophilic (55 °C) conditions. Statistical tests revealed no significant differences between both temperatures (see results and discussion section); thus, pretreated paper wastes were subjected to AD at 37 °C. Intermittent stirring, set at 112 rpm, was applied for 15 min daily to ensure adequate mixing in the reactors. Finally, the BMP from all reactors was calculated by subtracting the BMP measured in the control reactors which contained exclusively CS. An overview of all pretreated and non-pretreated paper wastes after 23 days of AD is presented in Table 2.

### Physico-chemical analyses

Physical and chemical parameters of fresh CS, WR and PR, as well as of digested sludges obtained after AD were measured. To determine TS, approximately 50 g of fresh sample was dried at 105 °C for 24 h and weighed after cooling in a desiccator. VS were calculated as the loss of weight after igniting 2 g of the oven-dried residue at 550 °C in a muffle furnace for 5 h. To extract total ammonia nitrogen (TAN), 15 ml of sample was mixed with 60 ml of a 0.0125 M calcium chloride solution and the mixture shaken at 150 rpm at room temperature for 1 h prior to being filtered through a paper filter. TAN concentration was measured using the colorimetric tube test (Macherey–Nagel GmbH and Co. KG, Düren, Germany) according to the manufacturer’s instructions. Free ammonia nitrogen (FAN) was calculated from TAN concentrations, as described in Calli et al. [49]. To determine pH of WR and PR, the residues were diluted 1:10 with distilled water and gently shaken at room temperature for 2 h. pH was measured with a portable multi-parameter meter Multi 340i (WTW, Weilheim, Germany). For high-performance liquid chromatography analyses, volatile fatty acids (VFAs) were extracted in dialysis tubing with 10 ml distilled water. Sample containing bottles were shaken three times and stored at 4 °C overnight in order to reach a total equilibrium in the dialysate. Dialysate (0.5 ml) was then transferred onto an Aminex HPX-87H column (Bio-Rad, Hercules, USA). A 5 mM H_2_SO_4_ mobile phase run at 0.7 ml min^−1^ and a detection wavelength of 210 nm were used to detect VFAs present at concentrations >0.5 mM.

### Microbiological analyses

#### DNA extraction

DNA extraction from the CS and all digested samples was conducted using the NucleoSpin^®^ Soil DNA isolation Kit (Macherey–Nagel GmbH and Co. KG, Düren, Germany) according to the manufacturer’s instructions. Quality of extracted DNA was controlled with agarose gel electrophoresis. Extracts were stored at −20 °C until use.

#### Dgge

DNA for DGGE analysis was amplified by the polymerase chain reaction (PCR). After an initial denaturation at 94 °C for 5 min, 33 cycles of 94 °C for 1 min, 49 °C for 2 min and 72 °C for 2 min were performed. An elongation step at 72 °C for 15 min completed DNA amplification. Each PCR reaction contained 0.2 µM of each primer (0357F-GC and 0691R; [50], 0.5 U µl^−1^ MyTaq DNA polymerase, 1 X MyTaq reaction buffer, 0.4 mg ml^−1^ bovine serum albumin (BSA) and 1 X enhancer (Peqlab, Germany). Finally, 1 µl extracted DNA was added to 24 µl of the PCR mastermix. PCR products were loaded into a 7–8 % (w/v) polyacrylamide gel with a denaturing gradient of 42–62 % (100 % denaturant consists of 7 M urea plus 40 % formamide in 1 X TAE buffer) and gels were run for 16 h at 100 V in 1 X TAE buffer (pH 7.4) at a constant temperature of 60 °C. Gels were stained with silver nitrate in an automated gel stainer (Amersham Pharmacia Biotech, Germany).

#### ***16S rRNA ***gene amplification and ANAEROCHIP hybridisation

DNA extracts of CS, WR37, PR37, WR55 and PR55 were subjected to PCR using the *16S rRNA* gene specific primers 109F and 934r [51]. PCR amplification, fluorescence labelling of target DNA, hybridisation, scanning and analysis of arrays were conducted as described by Franke-Whittle et al. [52]. SNR signals with intensities ≥2 were treated as positive signals.

#### Real-time quantitative PCR

To quantify methanogens detected using the ANAEROCHIP microarray, CS and the digested sludges from WR37, PR37, WR55, PR55, WR37us+en and PR37us+en were subjected to qPCR with genus-specific primers for *Methanosarcina*, *Methanobrevibacter*, *Methanoculleus* and *Methanosaeta*. Primers, as well as important standard curve parameters (efficiency, slope, intercept and *R*^*2*^) are presented in Table 3. qPCR was performed as described in Franke-Whittle et al. [52] by adding 2 µl of template DNA to a reaction volume of 18 µl. After an initial denaturation step at 95 °C for 5 min, thermal cycling comprised 45 cycles of 20 s at 95 °C, 20 s at 58–65 °C (annealing temperature) and 20 s at 72 °C. Annealing temperatures of 64 °C for *Methanosarcina*, 59 °C for *Methanobrevibacter*, 65 °C for *Methanoculleus* and 61 °C for *Methanosphaera* were used. In order to check for primer dimer formation and product specificity, thermal cycling was completed with a melting analysis (65–95 °C, ramp 0.5 °C min^−1^). Standard curves were constructed with PCR amplified *16S rDNA* from pure cultures of *Methanosarcina barkeri*, *Methanobrevibactersmithii*, *Methanoculleus* (Clone F_2FA36; [53] and *Methanosphaerastadtmanae*, as described in Franke-Whittle et al. [54] and Goberna et al. [33]. All standards and samples were run in duplicate.

#### Statistical analyses

The differences in CH_4_ production regarding both temperature (37 and 55 °C) and paper wastes type (WR and PR) were tested by non-parametric tests for pair-wise comparisons between different residues and temperature using the Mann–Whitney U test (p < 0.05), since data were not normally distributed.

To test the effects of the pretreatments (au, us, al, en, mo, us+en) on physico-chemical parameters and CH_4_ production on each of the paper wastes at 37 °C, data were subjected to a one-way ANOVA.

Methanogen quantification data (qPCR) were analysed by one-way ANOVA. The normality and the variance homogeneity of the data were tested prior to ANOVA. Data were transformed when they did not resemble a normal distribution. Significant differences in the main effects were analysed by paired comparisons with the Tukey HSD (honestly significant difference) as a post hoc test (p < 0.05). All statistical analyses were performed using the Statistica software program v9.

Comparison of archaeal DGGE patterns was conducted using the GelCompar II software package (Applied Maths, Belgium). DGGE bands were normalised using the reference position defined by the molecular-weight marker in order to align the bands for proper comparison. Cluster analysis was performed using Ochiai correlation coefficients and the Ward clustering algorithm. The programme settings were at 1 % optimisation and 1 % position tolerance.

PCA of SNRs from microarray data was conducted using CANOCO 5 [55].

## Abbreviations

AD: anaerobic digestion
Al: alkaline pretreatment
Au: autoclave pretreatment
BMP: biomethane potential
CS: cattle slurry
DGGE: denaturing gradient gel electrophoresis
En: enzyme pretreatment
FAN: free ammonia nitrogen
FW: fresh weight
GCN: gene copy numbers
Mo: commercial microorganism mixture addition
Nd: not detected
Nm: not measured
PR: pulp residues
qPCR: quantitative PCR
TAN: total ammonia nitrogen
TS: total solids
Us: ultrasound pretreatment
VFA: volatile fatty acids
VS: volatile solids
WR: wood residues
37: AD at 37 °C
55: AD at 55 °C
